# Temperament Traits and Chronic Pain: The Association of Harm Avoidance and Pain-Related Anxiety

**DOI:** 10.1371/journal.pone.0045672

**Published:** 2012-10-25

**Authors:** Peter Knaster, Ann-Mari Estlander, Hasse Karlsson, Jaakko Kaprio, Eija Kalso

**Affiliations:** 1 Department of Psychiatry, Helsinki University Central Hospital, Helsinki, Finland; 2 Pain Clinic, Helsinki University Central Hospital, Helsinki, Finland; 3 Hjelt Institute, Department of Public Health, University of Helsinki, Helsinki, Finland; Catholic University of Sacred Heart of Rome, Italy

## Abstract

**Objective:**

Anxiety symptoms are common in chronic pain patients. High levels of anxiety are associated with increased pain experience and disability. Proneness to anxiety has a large interindividual variation. The aim of the study was to determine whether the anxiety-related temperament trait Harm Avoidance (HA), is associated with pain-related anxiety.

**Methods:**

One hundred chronic pain patients in a multidisciplinary pain clinic participated in the study. The patients were assessed using the HA scale of the Temperament and Character Inventory (TCI) of Cloninger and Pain Anxiety Symptoms Scale-20 (PASS-20). Both the HA total score and the four subscales of HA were analyzed. Current pain intensity was measured using the Visual Analogue Scale (VAS). The Beck Depression Inventory (BDI) was used to control for the influence of depression on the personality measurement.

**Results:**

The HA total score was associated with PASS-20, but the association became non-significant after controlling for depression. The HA4 Fatigability subscale was associated with the PASS scales. Depression did not influence this association. Pain intensity was not correlated with HA or the PASS scales. However, the association between HA4 Fatigability and PASS was influenced by pain intensity. Higher pain intensity was associated with stronger association between the scales.

**Conclusion:**

Harm Avoidance, representing temperament and trait-related anxiety, has relevance in pain-related anxiety. Assessing personality and temperament may deepen the clinician's understanding of the pain experience and behavior in chronic pain patients.

## Introduction

Anxiety disorders are common in chronic pain patients. Anxiety-associated interpretations of pain, such as pain-catastrophizing, are important determinants of disability in pain patients. Excessive fear of pain contributes to physical inactivity and disuse which further worsen the disability and increase the pain experience [1]. In addition, pain-related catastrophic interpretations and avoidance behaviour may function as a risk factor in the development process from acute to chronic pain [1], [2].

Proneness to anxiety has a large interindividual variability. Personality related factors may partly explain the variation by enhancing vulnerability to anxiety. Among the most widely used personality models is the one presented by Robert Cloninger. According to the model, human personality is formed by temperament which is a biologically based emotional construct of personality, and character which represents a more mature personality part that develops through social learning and maturing processes. The temperamental traits are considered to be moderately heritable, present in early life, and have stability over the life span [3].

Harm Avoidance (HA) is a temperamental trait referring to a heritable tendency characterized by inhibition of behaviour as a response to signals of punishment and frustrative non-reward. HA is related to other personality associated constructs of negative affect such as neuroticism [4] and negative affectivity [5]. Several studies have shown the association between HA and depression [6]–[9] and anxiety disorders [9]–[13]. However, depressive patients have shown state dependent changes in HA scores indicating that HA may have both trait and state dependent characters [14]–[16]. Richter and colleagues reported that the two subscales of HA, the HA2 Fear of Uncertainty and the HA4 Fatigability were elevated in patients with recurrent depression suggesting their role as a possible risk factor for persistence of depression [17].

High levels of neuroticism have been associated with multiple somatic complaints and enhanced pain perception [18], [19] as well as pain-related fear and catastrophizing [20], [21]. Costa and McCrae have shown that negative affectivity correlates with health complaint scales and subjective distress rather than with the objective health status [22].

High Harm Avoidance has been related to heightened pain perception in healthy subjects in experimental studies [23], [24]. Furthermore, in recent studies chronic pain patients have had elevated levels of HA compared with healthy controls [25], [26]. However, chronic pain may have influence on personality measurements. Fishbain and colleagues reported that some of the trait scores measured by Minnesota Multiphasic Personality Inventory (MMPI) [27] changed with pain treatment denoting a state effect on personality measurement [28].

The role of the personality factors on pain-related anxiety is not well studied. Only a few studies [23]–[26], [29], [30] have used Cloninger's temperament model in pain patients. Our main hypothesis was that Harm Avoidance is associated to the more specific pain-related anxiety. A secondary hypothesis was that state effect of pain influences the association between HA and pain-related anxiety.

## Methods

### Patients

A total of 121 consecutive patients referred for assessment and treatment to the Helsinki University Central Hospital Pain Clinic were invited to participate in the study. Inclusion criteria were age from 30 to 60 years, chronic pain for at least one year, and fluency in the Finnish language. The exclusion criteria were malignancy, medication with strong opioids, psychosis, and current drug or alchohol abuse. Eighteen patients chose not to participate due to lack of interest or unknown reasons. Three patients were excluded due to the large amount of missing data. Thus, the study population consisted of 100 patients. The study was approved by the Ethics Committee of the Helsinki University Central Hospital. All patients provided a written informed consent.

### Data collection

The Pain Questionnaire, a routine self administered questionnaire used for all patients at the Helsinki University Central Hospital Pain Clinic was used. Demographic information and pain intensity measures using the Visual Analogue Scale (VAS), were extracted from this questionnaire. The patients were asked to mark on the line an estimate of their current pain intensity, the worst pain intensity during the past week, the mildest pain intensity during the past week, and current pain distress, using a 100 mm horizontal line with the 0 mm end representing no pain, and the 100 mm end representing maximum pain. As the other scales correlated (Pearson coefficients 0.553 to 0.706) with the current pain intensity, we decided to analyse only the current pain intensity.

In order to assess the possible state effect of depression the Beck Depression Inventory (BDI) [31], which is a self-administered scale measuring various symptoms of depression, was used. It is comprised of 21 groups of four statements describing the somatic and cognitive-affective symptoms of depression. The patients choose the alternative that best equals their state during the past week. A sum score is counted, a higher score indicating more severe depression. A number of studies support the validity and other psychometric properties of the BDI [32]–[35].

The Temperament and Character Inventory (TCI) [3], [36], [37] is a self administered questionnaire that is based on the psycobiological temperament model of Robert Cloninger. The 240 true/false question version was used. The factorial structure, internal validity and test-retest reliability of the TCI have been previously demonstrated in both general and psychiatric populations [37]–[41]. The Finnish version of the TCI was used [41]. In the present study we concentrated on the dimension of HA due to its relevance to the aims of the study.

The HA scale in the TCI is comprised of four subscales describing the different aspects of the trait:

Anticipatory Worry HA1 (11 items e.g. “Usually I am more worried than most people that something might go wrong in the future.”),
Fear of Uncertainty HA2 (7 items e.g. “I often feel tense and worried in unfamiliar situations, even when others feel there is little to worry about.”),
Shyness with Strangers HA3 (8 items e.g. “I often avoid meeting strangers because I lack confidence with people I do not know.”),

and Fatigability HA4 (9 items e.g. “I have less energy and get tired more quickly than most people.”).

The psychometric properties of the Finnish translation have been tested in a normal population sample of 4349 subjects. The results rendered support to the model with four temperament dimensions. The internal consistency of different HA subscales, measured by Cronbach α, varied between 0.64 and 0.72 [41].

Pain-related anxiety was assessed with the Pain Anxiety Symptom Scale-20 (PASS-20) [42], which is a shortened version of PASS [43]. It is comprised of 20 questions reflecting four facets of pain-related anxiety. Each item is measured by a 6-point Likert scale ranging from 0 = never to 5 = always.

The Fearfulness subscale describes fearful appraisals and interpretations about pain (e.g. “When I feel pain, I think I might be seriously ill.”). The Cognitive subscale describes cognitive anxiety and difficulties in concentrating (e.g.,“I can't think straight when in pain”.) The Escape/avoidance subscale describes avoidant reactions as a response to pain (e.g. “I go immediately to bed when I feel severe pain”), and the Physiological anxiety subscale describes physiological symptoms of anxiety (e.g. “Pain seems to cause my heart to pound and race”.) The factor structure presented by McCracken and Dhingra [42] was used in this study. PASS-20 has shown good reliability and validity, and is considered useful for both clinical and research applications [44]. The Finnish version of PASS-20 was used in the study. The Finnish version has previously been tested in a sample of 116 chronic pain patients. The mean score was 45.1 (SD 9.66, range 21–67). The Cronbach alpha for the total questionnaire was 0.94 [45].

### Statistical analysis

The statistical analyses were performed using the SPSS 19.0 for Windows. Means, standard deviations, distributions and frequencies for variables were calculated. For internal consistency the Cronbach α was used. The kurtosis, skewness, and normality of the continuous variables (Kolmogorov-Smirnov test) were assessed. The differences between genders were tested using Student's t-test.

Pearson correlation coefficient was used to calculate the association between the variables. A series of multiple regression analyses was performed to assess the association of the HA with pain-related anxiety. The dependent variables were PASS and each of its subscales. The independent variables were gender, age, current pain intensity, as well as the HA total score in the model one and the subscales of HA in the model two. Because of the one main outcome and four subscales a Bonferroni correction of 0.05/5 was used in the regression analysis.

The equations were reanalyzed after adding the BDI to the model. A small number of missing values were replaced by the mean of the variable. In order to identify possible multicollinearity, tolerance and the variance inflation factor (VIF) were calculated.

In order to study the effect of pain on the association, an interaction term was added to the equation. The pain severity level was considered as a moderator. The interaction was tested for those HA scales that had shown association with pain-related anxiety in the previous equations. The interaction term (pain severity×HA scale) was added after controlling the main effects of pain severity and the HA scale in question. The nature of interaction was studied visually by drawing regression lines representing the regression curves at +1SD, mean and −1 SD values of the pain variable [46].

## Results

Sixty-two percent of the patients were female. Mean age was 47.9 years (SD 7.32, range 30–60). Sixty percent were married or cohabiting. Twenty-five percent of the subjects had no professional education, 54% had a vocational education and 21% had a university level education. The employment status was the following: 39% were employed, 39% on sick leave, 12% on pension and 4% unemployed. The median duration of pain was 4 years (range 1–44 years). Sixty-one percent reported that they had had pain for 1–5 years; 22%, 5–10 years, and 16%, more than 10 years. The average current pain score was 59.8 mm (SD 21, range 0–100 mm) on the VAS scale. Men reported higher mean pain intensity than women, (p = 0.013, t = 2.47). This was the only variable with a significant difference between the genders. Descriptive statistics of the pain measure and psychological variables are presented in table 1.

**Table 1:** Descriptive statistics of the psychological values and the pain measurement.

	Minimum	Maximum	Mean	Std. Deviation	Cronbach alpha
Harm Avoidance score (HA)	2.0	34.0	17.0	6.9	0.88
HA1 Anticipatory Worry	1.0	11.0	4.8	2.5	0.73
HA2 Fear of Uncertainty	.0	7.0	3.7	1.9	0.66
HA3 Shyness with Strangers	.0	8.0	2.9	2.2	0.75
HA4 Fatigability	.0	9.0	5.7	2.2	0.72
PASS total	8.0	95.0	47.4	17.9	0.91
Fearfulness	.0	22.0	10.2	5.3	0.77
Escape avoidance	2.0	24.0	12.5	5.2	0.76
Cognitive anxiety	4.0	25.0	15.5	5.1	0.82
Physiological anxiety	.0	25.0	9.2	5.3	0.72
Beck Depression Inventory score	1.0	46.0	17.4	10.3	0.90
VAS current pain 0–100 mm	.0	100.0	59.8	21.2	-

The patients in the study did not differ significantly from those 18 patients who chose not to participate, regarding the mean age or gender distribution. The number of the drop outs was small (3), and they were estimated not to affect the results..

Forty-nine percent of patients were classified as having neuropathic pain, 21% had nociceptive pain, 5% visceral pain, and 25% had idiopathic pain. The most common pain etiologies were arthrotic/connective tissue 20%, spinal cord/spinal root/prolapsed disc 19%, chronic pain without known origin 18%, traumatic peripheral neuropathy 11%, other peripheral neuropathy 8%, and fibromyalgia 4%.

All the continuous variables except HA3 (Shyness with Strangers) (Z = 1, 610, p = 0.011) had normal distributions according to the Kolmogorov-Smirnov test. As the kurtosis and skewness values of HA3 were acceptable, it was accepted in the analysis. After substituting the missing values of the independent variables one patient was excluded because of a missing value of the dependent variable (PASS).

The correlations between the variables are presented in table 2. No statistically significant correlations between pain intensity and any of the HA or PASS scales were found. Inter-correlations existed between the PASS subscales as expected. The HA subscales were also inter-correlated although the level was not as strong.

**Table 2:** Zero-order Pearson correlation coefficients for the pain measure and the psychological variables.

	PASS total	PASS Fearf.	PASS Esc/avoid.	PASS Cogn.	PASS Phys.	Harm Avoidance score	HA1 Anticip. Worry	HA2 Fear of Uncert.	HA3 Shyness with Str.	HA4 Fatig.	BDI
Current pain	.041	.014	−.010	−.075	.202	.194	.062	.192	.190	.179	.149
PASS total	1	.866*	.867*	.835*	.812*	.322*	.337*	.054	.109	.480*	.425*
PASS Fearfulness		1	.662*	.662*	.594*	.342*	.421*	.095	.129	.394*	.387*
PASS Escape/avoidance			1	.656*	.618*	.263	.201	.075	.103	.435*	.266
PASS Cognitive				1	.522*	.158	.191	−.067	.009	.331*	.309*
PASS Physiological					1	.318*	.317*	.072	.125	.457*	.468*
Harm Avoidance score						1	.841*	.765*	.797*	.729*	.463*
HA1 Anticipatory Worry							1	.504*	.539*	.540*	.429*
HA2 Fear of Uncertainty								1	.600*	.364*	.062
HA3 Shyness with Strangers									1	.365*	.297
HA4 Fatigability										1	.541*
BDI											1

* Pearson correlation coefficient >0.3, significant at the 0.01 level (2-tailed).

Positive correlations existed between several HA and PASS scores. The HA4 Fatigability score correlated with each of the PASS scores, correlations ranging from r = 0.331 (PASS Cognitive) to r = 0.480 (PASS total). The HA1 Anticipatory Worry had positive correlations with PASS scores whereas the two remaining subscales HA2 Fear of Uncertainty and HA3 Shyness with Strangers did not correlate with any of the PASS scores. The BDI score had weak or moderate positive correlation coefficients with several PASS and HA scales.

**Figure 1:**
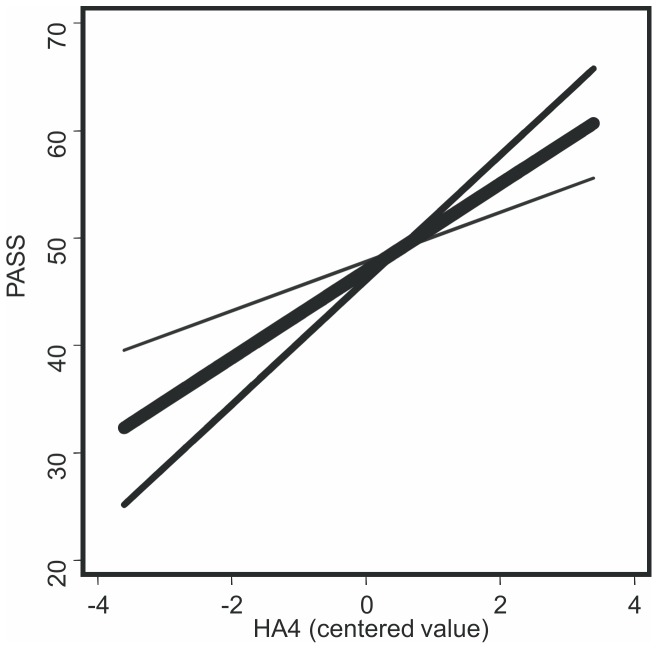
Influence of pain intensity on the relationship between Harm Avoidance Fatigability HA4 -subscale and pain-related anxiety.

In the regression analyses the variance-inflation factors were acceptable, 1.038–2.035, indicating that the multicollinearity problem did not exist. In the first model the HA total score was significantly associated with PASS and its subscales except the PASS cognitive subscale (p = 0.098) (Table S1). After adding the BDI variable to the equation, these associations became non-significant (Table 3.). In the second model the HA4 Fatigability subscale had a significant association with all PASS scores except PASS Fearfulness, which was associated with the HA1 Anticipatory Worry scale (Table S1). Adding the BDI to the equation altered HA4 Fatigability associations only very little (Table 4.). As the HA4 Fatigability scale appeared significant, an interaction variable (HA4 Fatigability×pain intensity) was added to a regression model after gender, age, and pain intensity and HA subscales. The interaction term was a significant (B = 0.83, p = 0.020) predictor revealing that the association between HA4 Fatigability and pain-related anxiety was conditional on the level of pain. Adding the interaction term increased the explained variance by 0.044 (F change 5.74, df 1,88, p = 0.019). The interaction term remained significant (B = 0.86, p = 0.031) even after controlling for the effect of depressive state (BDI). Patients with a pain level 1 SD above the mean had a stronger association (B = 5.98, p<0.001), compared to those having 1 SD below the mean (B = 2.26, p = 0.024). The interaction was further visualized in the plots indicating that the association was more pronounced if pain intensity was high (Figure 1.). The two other interaction terms (HA total score×pain intensity and HA1 Anticipatory Worry×pain intensity), remained non-significant. In all equation models pain intensity remained unassociated with PASS and its subscales.

**Table 3:** Multiple regression analyses with PASS scales as dependent variables and Harm Avoidance (HA) as an independent variable.

	PASS total			PASS Fear			PASS Escape/Avoid.			PASS Cogn.			PASS Physiol.		
Model 1	β^a^	t	p	β^a^	t	p	β^a^	t	p	β^a^	t	p	β^a^	t	p
Gender	−.005	−.051	.960	.015	.158	.875	−.064	−.628	.523	.070	.688	.493	−.035	−.375	.708
Age	.034	.342	.733	.089	.899	.371	.024	.231	.818	.029	.277	.782	−.029	−.300	.765
Current pain	−.059	−.579	.564	−.096	−.943	.348	−.101	−.944	.348	−.122	1.144	.256	.115	1.172	.244
HA	.163	1.525	.131	.206	1.927	.057	.193	1.708	.091	.028	.251	.803	.116	1.124	.264
BDI	.362	3.389	.001	.316	2.939	.004	.196	1.728	.087	.318	2.828	.006	.392	3.775	<.001
Full model	Adj R^2^			Adj R^2^			Adj R^2^			Adj R^2^			Adj R^2^		
	.161			.152			.058			.070			.206		

a standardized coefficient results with p<0.01 are considered significant (Bonferroni adjustment 0.05/5).

**Table 4:** Multiple regression analyses with PASS scales as dependent variables and the subscales of HA as independent variables.

	PASS total			PASS Fear			PASS Escape/Avoid.			PASS Cogn.			PASS Physiol.		
Model 2	β^a^	t	p	β^a^	t	p	β^a^	t	p	β^a^	t	p	β^a^	t	p
Gender	−.001	−.010	.992	−.008	−.088	.930	−.036	−.358	.721	.082	.813	.418	−.037	−.394	.695
Age	.054	.569	.571	.073	.748	.456	.074	.723	.471	.056	.542	.589	−.017	−.178	.859
Current pain	−.050	−.507	.614	−.060	−.594	.554	−.122	−1.168	.246	−.118	−1.119	.266	.124	1.267	.208
HA1 Anticipatory Worry	.153	1.149	.254	.345	2.548	.013	−.034	−.241	.810	.060	.418	.677	.134	1.011	.315
HA2 Fear of Uncertainty	−.121	−.975	.332	−.098	−.777	.439	−.063	−.481	.632	−.180	−1.351	.180	−.071	−.576	.566
HA3 Shyness with Strangers	−.108	−.901	.370	−.136	−1.105	.272	−.027	−.210	.835	−.077	−.597	.552	−.124	−1.032	.305
HA4 Fatigability	.391	3.312	.001	.223	1.853	.067	.479	3.821	<.001	.338	2.669	.009	.288	2.453	.016
BDI	.196	1.650	.103	.165	1.359	.178	.066	.521	.603	.160	1.253	.214	.270	2.277	.025
Full model	Adj R^2^			Adj R^2^			Adj R^2^			Adj R^2^			Adj R^2^		
	.239			.208			.144			.127			.246		

a standardized coefficient results with p<0.01 are considered significant (Bonferroni adjustment 0.05/5).

## Discussion

In the present study the HA of Cloninger's TCI, reflecting the biological tendency characterized by behavioral inhibition, showed a positive association with pain-related anxiety. The HA4 Fatigability score showed the strongest linkage with anxiety having an association with each of the PASS scales. Also the HA1 Anticipatory Worry score was related to anxiety, but only to the PASS fearfulness scale. In the correlation analyses the associations between current pain intensity and HA or anxiety measures remained non-significant. However, an interaction between pain intensity and HA4 Fatigability score was present, indicating that patients with more severe pain had stronger association between HA4 and PASS compared to those with less severe pain.

After controlling the state effect of depression measured by BDI, the associations between the HA total score and PASS scales became non-significant. However, the adding the BDI variable to the equations did not remarkably affect the association between the HA4 Fatigability and PASS scales, nor its interaction effect with pain intensity on PASS.

The association of HA and anxiety agrees with the results of previous studies performed among psychiatric patients with anxiety disorders [11], [47]. The association has also been present in a general population [48] as well as in non-clinical samples [12], [49]. The role of HA as a vulnerability factor for anxiety disorders has been unclear. High HA scores have been reported in the relatives of individuals having the obsessive compulsive disorder (OCD) suggesting that HA might have a role as a familial risk factor [50]. Harm Avoidance has also been linked to anxiety sensitivity [51], an anxiety-related construct [52], which has been presented as a predictor of pain-related fear and anxiety [53].

In the present study the association between PASS and HA relied mainly on the Fatigability –subscale and to a lesser extent on the Anticipatory Worry subscale. The third subscale, Shyness with Strangers, involves statements concerning social avoidance. Thus, a linkage between that subscale and pain-related anxiety scales is understandably weak. The other unassociated HA scale, Fear of Uncertainty, describes tendencies to avoid risk situations. The fear-related questions in PASS concentrate mainly on the fearful appraisals of pain instead of assessing risks, which may explain the discrepancy. The HA Fatigability scale showed the clearest association with the PASS scales. According to the model of Cloninger individuals with high Fatigability have low energy level depending on their personality characteristics. They recover from minor illnesses and stress more slowly than average people do [37]. Fatigability as a trait construct reflects the negative affectivity associated with enhanced subjective distress in somatic illnesses. Persons with high level of neuroticism tend to focus their attention on internal somatic sensations and give them negative interpretations [18]. Thus also individuals with high level of HA indicating pessimism, worries, fear, and passive tendencies, may have a reduced threshold to experience more pain-related stress than those with low level of HA.

Personality trait measures may change in chronic pain patients with pain treatment indicating state dependence of the measurements [28]. Fatigue and insomnia are common symptoms in chronic pain and other somatic illnesses. As several items of the HA4 Fatigability subscale describe energy level and tiredness, it is possible that the state contamination effect interferes with the measurement. However, previous temperament studies in chronic pain patients have shown higher scores in the HA4 and HA1 subscales compared to pain-free controls while the other HA subscales have not shown a difference [25], [26]. Whether this finding is personality related or state dependent is unclear, because of the cross sectional study design.

The association between HA and PASS became non-significant after controlling for the BDI, suggesting a confounding effect of the depressive state. This may reflect the state effect of depression affecting the trait measurement. [54]. Because of the several somatic items of BDI, there have been concerns about criterion contamination and the validity of the scale [55]. Morley and colleagues have suggested that depression in chronic pain patients differs from the psychiatric model of depression and recommended the use of a specific factor model of BDI in pain patients [56]. It is possible that part of the confounding effect of BDI is due to these somatic or unspecific symptoms.

The pain intensity level affected the relation between the HA4 Fatigability scale and pain-related anxiety. Patients who experienced higher pain level had stronger association between fatigability and anxiety, compared to those who experienced low pain level. The state of depression did not influence this relation. Because of the cross sectional study design the interpretations of this finding are hypothetical. The pain measurement with VAS measures the subjective experience of pain which is linked to several external and internal factors. A higher level of pain can cause more anxiety in persons who are constitutionally low in energy and easily tired. Because the interaction was present only in the Fatigability scale, the possibility of a state effect of pain cannot be eliminated.

The cross-sectional study design is a major limitation of the study, preventing any causality judgements. Therefore, the results need to be tested prospectively with repeated assessments of pain and anxiety in the same patients over time.

Further limitations of the study are the relatively small number of patients, the lack of a control group, and the reliance on self-report data. The choice to analyse only HA and exclude the other three temperament scales can also be criticized. However, HA can be considered the most relevant temperament scale regarding pain-related anxiety. In addition, the pain measurement was limited to current pain only. The pain questionnaire used in the study included also the visual analogue scales measuring the “pain at worst”, “pain at best”, and “pain distress”. However, additional information was limited due to high intercorrelations and these scales were omitted. Considering the long duration of chronic pain, the patients are likely to recollect the current pain intensity measure most accurately. The heterogeneous pain disorders of the patients may also complicate the interpretation of the results. The HA scores may be susceptible to demographic factors such as gender, age, and educational level. The patient sample in a tertiary clinic is also highly selected. Thus the results of the study are not to be generalized to all chronic pain patients.

In conclusion, specific aspects of Harm Avoidance have relevance to pain-related anxiety. The HA measurement is susceptible to the state effect of depression, however it may explain part of the interindividual variation in pain-related fear and avoidance behaviour. The association between HA and pain-related anxiety may reflect both trait, a general tendency, and state, a situation-related phenomenon. Prospective studies would further clarify the role of HA as a vulnerability factor in chronic pain and pain-related anxiety.

In clinical practice, assessing temperament may help to understand the individual's experience of pain and the related pain behaviour. The anxiety level of the patients affects broadly the whole treatment process. Patients with avoidant or passive reaction styles are likely to need more supportive and intensive treatment methods. Trait anxiety may have an even more profound effect, because of its stability and more constant nature. In the future longitudinal studies could clarify the role of temperamental factors in pain-related anxiety and pain pathogenesis in general.

## Supporting Information

Dataset S1 The data for pain intensity, pain-related anxiety, and harm avoidance scores of the chronic pain patients.
(XLS)

Table S1 Multiple regression analyses with PASS scales as dependent variables and HA scales as independent variables, with and without BDI as a control variable.
(DOC)

## References

[1] Vlaeyen JW, Linton SJ (2000) Fear-avoidance and its consequences in chronic musculoskeletal pain: A state of the art. Pain 85 3: 317–332. 10781906 10.1016/S0304-3959(99)00242-0

[2] Lethem J, Slade PD, Troup JD, Bentley G (1983) Outline of a fear-avoidance model of exaggerated pain perception–I. Behav Res Ther 21 4: 401–408. 6626110 10.1016/0005-7967(83)90009-8

[3] Cloninger CR, Svrakic DM, Przybeck TR (1993) A psychobiological model of temperament and character. Arch Gen Psychiatry 50 12: 975–990. 8250684 10.1001/archpsyc.1993.01820240059008

[4] McCrae RR, Costa PT Jr (1987) Validation of the five-factor model of personality across instruments and observers. J Pers Soc Psychol 52 1: 81–90. 3820081 10.1037//0022-3514.52.1.81

[5] Watson D, Clark LA (1984) Negative affectivity: The disposition to experience aversive emotional states. Psychol Bull 96 3: 465–490. 6393179

[6] Hansenne M, Reggers J, Pinto E, Kjiri K, Ajamier A, et al (1999) Temperament and character inventory (TCI) and depression. J Psychiatr Res 33 1: 31–36. 10094237 10.1016/s0022-3956(98)00036-3

[7] Naito M, Kijima N, Kitamura T (2000) Temperament and character inventory (TCI) as predictors of depression among japanese college students. J Clin Psychol 56 12: 1579–1585. 11132572 10.1002/1097-4679(200012)56:12<1579::AID-8>3.0.CO;2-K

[8] Elovainio M, Kivimaki M, Puttonen S, Heponiemi T, Pulkki L, et al (2004) Temperament and depressive symptoms: A population-based longitudinal study on cloninger's psychobiological temperament model. J Affect Disord 83 2–3: 227–232. 15555718 10.1016/j.jad.2004.06.005

[9] Jylha P, Isometsa E (2006) The relationship of neuroticism and extraversion to symptoms of anxiety and depression in the general population. Depress Anxiety 23 5: 281–289. 16688731 10.1002/da.20167

[10] Svrakic DM, Przybeck TR, Cloninger CR (1992) Mood states and personality traits. J Affect Disord 24 4: 217–226. 1578077 10.1016/0165-0327(92)90106-g

[11] Marteinsdottir IMD, Tillfors MPD, Furmark TPD, Anderberg UMMD, Ekselius LMD (2003) Personality dimensions measured by the temperament and character inventory (TCI) in subjects with social phobia.[article]. Nordic Journal of Psychiatry 57 1: 29–35. 12745789 10.1080/08039480310000239

[12] Matsudaira T, Kitamura T (2006) Personality traits as risk factors of depression and anxiety among japanese students. J Clin Psychol 62 1: 97–109. 16287151 10.1002/jclp.20215

[13] Ettelt S, Grabe HJ, Ruhrmann S, Buhtz F, Hochrein A, et al (2008) Harm avoidance in subjects with obsessive-compulsive disorder and their families. J Affect Disord 107 1–3: 265–269. 17854908 10.1016/j.jad.2007.08.017

[14] Farmer A, Mahmood A, Redman K, Harris T, Sadler S, et al (2003) A sib-pair study of the temperament and character inventory scales in major depression. Arch Gen Psychiatry 60 5: 490–496. 12742870 10.1001/archpsyc.60.5.490

[15] Farmer RF, Seeley JR (2009) Temperament and character predictors of depressed mood over a 4-year interval. Depress Anxiety 26 4: 371–381. 19123455 10.1002/da.20459

[16] Nery FG, Hatch JP, Nicoletti MA, Monkul ES, Najt P, et al (2009) Temperament and character traits in major depressive disorder: Influence of mood state and recurrence of episodes. Depress Anxiety 26 4: 382–388. 19195006 10.1002/da.20478

[17] Richter J, Eisemann M, Richter G (2000) Temperament and character during the course of unipolar depression among inpatients. Eur Arch Psychiatry Clin Neurosci 250 1: 40–47. 10738864 10.1007/pl00007538

[18] Watson D, Pennebaker JW (1989) Health complaints, stress, and distress: Exploring the central role of negative affectivity. Psychol Rev 96 2: 234–254. 2710874 10.1037/0033-295x.96.2.234

[19] Ramirez-Maestre C, Lopez Martinez AE, Zarazaga RE (2004) Personality characteristics as differential variables of the pain experience. J Behav Med 27 2: 147–165. 15171104 10.1023/b:jobm.0000019849.21524.70

[20] Muris P, Meesters C, van den Hout A, Wessels S, Franken I, et al (2007) Personality and temperament correlates of pain catastrophizing in young adolescents. Child Psychiatry Hum Dev 38 3: 171–181. 17406972 10.1007/s10578-007-0054-9 PMC2778719

[21] Goubert L, Crombez G, Van Damme S (2004) The role of neuroticism, pain catastrophizing and pain-related fear in vigilance to pain: A structural equations approach. Pain 107 3: 234–241. 14736586 10.1016/j.pain.2003.11.005

[22] Costa PT Jr, McCrae RR (1987) Neuroticism, somatic complaints, and disease: Is the bark worse than the bite?. J Pers 55 2: 299–316. 3612472 10.1111/j.1467-6494.1987.tb00438.x

[23] Pud D, Eisenberg E, Sprecher E, Rogowski Z, Yarnitsky D (2004) The tridimensional personality theory and pain: Harm avoidance and reward dependence traits correlate with pain perception in healthy volunteers. European Journal of Pain: Ejp 8 1: 31–38. 14690672 10.1016/S1090-3801(03)00065-X

[24] Granot M (2005) Personality traits associated with perception of noxious stimuli in women with vulvar vestibulitis syndrome. J Pain 6 3: 168–173. 15772910 10.1016/j.jpain.2004.11.010

[25] Malmgren-Olsson EB, Bergdahl J (2006) Temperament and character personality dimensions in patients with nonspecific musculoskeletal disorders. Clin J Pain 22 7: 625–631. 16926578 10.1097/01.ajp.0000210907.65170.a3

[26] Conrad R, Schilling G, Bausch C, Nadstawek J, Wartenberg HC, et al (2007) Temperament and character personality profiles and personality disorders in chronic pain patients. Pain 133 1–3: 197–209. 17964076 10.1016/j.pain.2007.07.024

[27] Hathaway SR, McKinley JC (2001) Minnesota multiphasic personality inventory (MMPI, MMPI-2 and MMPI-A). In: Anonymous American Psychiatric Association Handbook of Psychiatric Measures. Washington DC: Am Psychiatric Association Press. pp. 89–92.

[28] Fishbain DA, Cole B, Cutler RB, Lewis J, Rosomoff HL, et al (2006) Chronic pain and the measurement of personality: Do states influence traits? Pain Med 7 6: 509–529. 17112364 10.1111/j.1526-4637.2006.00239.x

[29] Boz C, Velioglu S, Ozmenoglu M, Sayar K, Alioglu Z, et al (2004) Temperament and character profiles of patients with tension-type headache and migraine. Psychiatry & Clinical Neurosciences 58 5: 536–543. 15482586 10.1111/j.1440-1819.2004.01297.x

[30] Mongini F, Fassino S, Rota E, Deregibus A, Levi M, et al (2005) The temperament and character inventory in women with migraine. J Headache Pain 6 4: 247–249. 16362677 10.1007/s10194-005-0198-6 PMC3452018

[31] Beck AT, Ward CH, Mendelson M, Mock J, Erbaugh J (1961) An inventory for measuring depression. Arch Gen Psychiatry 4: 561–571. 13688369 10.1001/archpsyc.1961.01710120031004

[32] Kearns NP, Cruickshank CA, McGuigan KJ, Riley SA, Shaw SP, et al (1982) A comparison of depression rating scales. Br J Psychiatry 141: 45–49. 7116071 10.1192/bjp.141.1.45

[33] Beck AT, Steer RA (1984) Internal consistencies of the original and revised beck depression inventory. J Clin Psychol 40 6: 1365–1367. 6511949 10.1002/1097-4679(198411)40:6<1365::aid-jclp2270400615>3.0.co;2-d

[34] Varjonen J, Romanov K, Kaprio J, Heikkila K, Koskenvuo M (1997) Self-rated depression in 12,063 middle-aged adults. Nordic Journal of Psychiatry 51 5: 331–338.

[35] Steer RA, Cavalieri TA, Leonard DM, Beck AT (1999) Use of the beck depression inventory for primary care to screen for major depression disorders. Gen Hosp Psychiatry 21 2: 106–111. 10228890 10.1016/s0163-8343(98)00070-x

[36] Cloninger CR (1987) A systematic method for clinical description and classification of personality variants. A proposal. Arch Gen Psychiatry 44 6: 573–588. 3579504 10.1001/archpsyc.1987.01800180093014

[37] Cloninger CR, Przybeck TR, Svrakic DM, Wetzel RD (1994) The temperament and character inventory (TCI): A guide to its development and use. St Louis, Missouri: Center for Psychobiology of Personality, Washington University.

[38] Brandstrom S, Schlette P, Przybeck TR, Lundberg M, Forsgren T, et al (1998) Swedish normative data on personality using the temperament and character inventory. Compr Psychiatry 39 3: 122–128. 9606577 10.1016/s0010-440x(98)90070-0

[39] Brändström S, Richter J, Przybeck T (2001) Distributions by age and sex of the dimensions of temperament and character inventory in a cross-cultural perspective among sweden, germany, and the USA. Psychol Rep 89 3: 747–758. 11824747 10.2466/pr0.2001.89.3.747

[40] Pelissolo A, Lepine JP (2000) Normative data and factor structure of the temperament and character inventory (TCI) in the french version. Psychiatry Res 94 1: 67–76. 10788679 10.1016/s0165-1781(00)00127-x

[41] Miettunen J, Kantojarvi L, Ekelund J, Veijola J, Karvonen JT, et al (2004) A large population cohort provides normative data for investigation of temperament. Acta Psychiatr Scand 110 2: 150–157. 15233716 10.1111/j.1600-0047.2004.00344.x

[42] McCracken LM, Dhingra L (2002) A short version of the pain anxiety symptoms scale (PASS-20): Preliminary development and validity. Pain Res Manag 7 1: 45–50. 16231066 10.1155/2002/517163

[43] McCracken LM, Zayfert C, Gross RT (1992) The pain anxiety symptoms scale: Development and validation of a scale to measure fear of pain. Pain 50 1: 67–73. 1513605 10.1016/0304-3959(92)90113-P

[44] Coons MJ, Hadjistavropoulos HD, Asmundson GJG (2004) Factor structure and psychometric properties of the pain anxiety symptoms scale-20 in a community physiotherapy clinic sample. European Journal of Pain 8 6: 511–516. 15531218 10.1016/j.ejpain.2003.11.018

[45] Estlander AM Unpublished material.

[46] Aiken LS, West SG (1991) Multiple regression: Testing and interpreting interactions. London, United Kingdom: SAGE Publications, Inc.

[47] Faytout M, Tignol J, Swendsen J, Grabot D, Aouizerate B, et al (2007) Social phobia, fear of negative evaluation and harm avoidance. Eur Psychiatry 22 2: 75–79. 17101266 10.1016/j.eurpsy.2005.07.009

[48] Jylha P, Isometsa E (2006) Temperament, character and symptoms of anxiety and depression in the general population. Eur Psychiatry 21 6: 389–395. 16360306 10.1016/j.eurpsy.2005.09.003

[49] Jiang N, Sato T, Hara T, Takedomi Y, Ozaki I, et al (2003) Correlations between trait anxiety, personality and fatigue: Study based on the temperament and character inventory. J Psychosom Res 55 6: 493–500. 14642978 10.1016/s0022-3999(03)00021-7

[50] Ettelt S, Grabe HJ, Ruhrmann S, Buhtz F, Hochrein A, et al (2008) Harm avoidance in subjects with obsessive-compulsive disorder and their families. J Affect Disord 107 1–3: 265–269. 17854908 10.1016/j.jad.2007.08.017

[51] Van der Does W, Duijsens I, Eurelings-Bontekoe E, Verschuur M, Spinhoven P (2003) Anxiety sensitivity profile: Dimensional structure and relationship with temperament and character. Psychother Psychosom 72 4: 217–222. 12792127 10.1159/000070786

[52] Reiss S, McNally RJ (1985) The expectancy model of fear. In: Reiss S, Bootzin RR, editors. Theoretical issues in behavioral therapy. New York: Academic Press. pp. 107–121.

[53] Zvolensky MJ, Goodie JL, McNeil DW, Sperry JA, Sorrell JT (2001) Anxiety sensitivity in the prediction of pain-related fear and anxiety in a heterogeneous chronic pain population. Behaviour Research & Therapy 39 6: 683–696. 11400712 10.1016/s0005-7967(00)00049-8

[54] Farmer A, Redman K, Harris T, Mahmood A, Sadler S, et al (2002) Neuroticism, extraversion, life events and depression. the cardiff depression study. Br J Psychiatry 181: 118–122. 12151281 10.1017/s0007125000161823

[55] Pincus T, Williams A (1999) Models and measurements of depression in chronic pain. J Psychosom Res 47 3: 211–219. 10576470 10.1016/s0022-3999(99)00045-8

[56] Morley S, Williams AC, Black S (2002) A confirmatory factor analysis of the beck depression inventory in chronic pain. Pain 99 1–2: 289–298. 12237207 10.1016/s0304-3959(02)00137-9

