# Supplementary material for: Temperament Traits and Chronic Pain: The Association of Harm Avoidance and Pain-Related Anxiety
Source: PLoS One. 2012 Oct 25;7(10):e45672. doi: 10.1371/journal.pone.0045672 (PMC3485083; doi:10.1371/journal.pone.0045672)
Supplement: Table S1 — Multiple regression analyses with PASS scales as dependent variables and HA scales as independent variables, with and without BDI as a control variable. (DOC) [file pone.0045672.s002.doc]

|  | PASS  total |  |  | PASS  Fearfulness |  |  | PASS  Escape/  avoidance |  |  | PASS  Cognitive |  |  | PASS  Physiological |  |  |
| --- | --- | --- | --- | --- | --- | --- | --- | --- | --- | --- | --- | --- | --- | --- | --- |
| Model 1 | βª | t | p | βª | t | p | βª | t | p | βª | t | p | βª | t | p |
| Gender | -.009 | -.087 | .931 | .012 | .118 | .906 | -.066 | -.642 | .523 | .067 | .631 | .530 | -.040 | -.394 | .694 |
| Age | -.030 | -.290 | .773 | .034 | .335 | .738 | -.010 | -.097 | .923 | -.027 | -.254 | .800 | -.097 | -.968 | .336 |
| Current pain | -.016 | -.155 | .877 | -.059 | -.563 | .575 | -.078 | -.729 | .468 | -.085 | -.773 | .441 | .161 | 1.544 | .126 |
| Harm Avoidance (HA) | .328 | 3.278 | .001 | .350 | 3.535 | .001 | .282 | 2.779 | .007 | .173 | 1.674 | .098 | .295 | 2.999 | .003 |
|  | Adj R² |  |  | Adj R² |  |  | Adj R² |  |  | Adj R² |  |  | Adj R² |  |  |
| Full model^b^ | .067 |  |  | .083 |  |  | .038 |  |  | .001 |  |  | .094 |  |  |
|  |  |  |  |  |  |  |  |  |  |  |  |  |  |  |  |
| Gender | -.005 | -.051 | .960 | .015 | .158 | .875 | -.064 | -.628 | .532 | .070 | .688 | .493 | -.035 | -.375 | .708 |
| Age | .  .034 | .342 | .733 | .089 | .899 | .371 | .024 | .231 | .818 | .029 | .277 | .782 | -.029 | -.300 | .765 |
| Current pain | -.059 | -.579 | .564 | -.096 | -.943 | .348 | -.101 | -.944 | .348 | -.122 | -1.144 | .256 | .115 | 1.172 | .244 |
| Harm Avoidance (HA) | .163 | 1.525 | .131 | .206 | 1.927 | .057 | .193 | 1.708 | .091 | .028 | .251 | .803 | .116 | 1.124 | .264 |
| BDI | .362 | 3.389 | .001 | .316 | 2.939 | .004 | .196 | 1.728 | .087 | .318 | 2.828 | .006 | .392 | 3.775 | <.001 |
|  | Adj R² |  |  | Adj R² |  |  | Adj R² |  |  | Adj R² |  |  | Adj R² |  |  |
| Full model | .161 |  |  | .152 |  |  | .058 |  |  | .070 |  |  | .206 |  |  |

Table S1. Multiple regression analyses with PASS scales as dependent variables and HA scales as independent variables, with and without BDI as a control variable.

|  | PASS  total |  |  | PASS  Fearfulness |  |  | PASS  Escape/  avoidance |  |  | PASS  Cognitive |  |  | PASS  Physiological |  |  |
| --- | --- | --- | --- | --- | --- | --- | --- | --- | --- | --- | --- | --- | --- | --- | --- |
| Model 2 | βª | t | p | βª | t | p | βª | t | p | βª | t | p | βª | t | p |
| Gender | -.003 | -.029 | .977 | -.010 | -.104 | .917 | -.036 | -.366 | .715 | .080 | .796 | .428 | -.039 | -.412 | .681 |
| Age | .031 | .321 | .749 | .053 | .547 | .586 | .066 | .655 | .514 | .036 | .356 | .722 | -.050 | -.515 | .608 |
| Current pain | -.024 | -.246 | .806 | -.038 | -.382 | .703 | -.113 | -1.103 | .273 | -.097 | -.929 | .355 | .159 | 1.612 | .110 |
| HA1 Anticipatory Worry | .231 | 1.843 | .069 | .411 | 3.232 | .002 | -.008 | -.059 | .953 | .123 | .923 | .358 | .241 | 1.909 | .059 |
| HA2 Fear of Uncertainty | -.194 | -1.663 | .100 | -.160 | -1.348 | .181 | -.088 | -.718 | .474 | -.239 | -1.923 | .058 | -.172 | -1.459 | .148 |
| HA3 Shyness with Strangers | -.078 | -.648 | .519 | -.110 | -.902 | .369 | -.016 | -.131 | .896 | -.052 | -.407 | .685 | -.082 | -.673 | .502 |
| HA4 Fatigability | .465 | 4.208 | <.001 | .285 | 2.542 | .013 | .503 | 4.356 | <.001 | .397 | 3.382 | .001 | .389 | 3.497 | .001 |
|  | Adj R² |  |  | Adj R² |  |  | Adj R² |  |  | Adj R² |  |  | Adj R² |  |  |
| Full model | .225 |  |  | .200 |  |  | .151 |  |  | .122 |  |  | .211 |  |  |
|  |  |  |  |  |  |  |  |  |  |  |  |  |  |  |  |
| Gender | -.001 | -.010 | .992 | -.008 | -.088 | .930 | -.036 | -.358 | .721 | .082 | .813 | .418 | -.037 | -.394 | .695 |
| Age | .054 | .569 | .571 | .073 | .748 | .456 | .074 | .723 | .471 | .056 | .542 | .589 | -.017 | -.178 | .859 |
| Current pain | -.050 | -.507 | .614 | -.060 | -.594 | .554 | -.122 | -1.168 | .246 | -.118 | -1.119 | .266 | .124 | 1.267 | .208 |
| HA1 Anticipatory Worry | .153 | 1.149 | .254 | .345 | 2.548 | .013 | -.034 | -.241 | .810 | .060 | .418 | .677 | .134 | 1.011 | .315 |
| HA2 Fear of Uncertainty | -.121 | -.975 | .332 | -.098 | -.777 | .439 | -.063 | -.481 | .632 | -.180 | -1.351 | .180 | -.071 | -.576 | .566 |
| HA3 Shyness with Strangers | -.108 | -.901 | .370 | -.136 | -1.105 | .272 | -.027 | -.210 | .835 | -.077 | -.597 | .552 | -.124 | -1.032 | .305 |
| HA4 Fatigability | .391 | 3.312 | .001 | .223 | 1.853 | .067 | .479 | 3.821 | <.001 | .338 | 2.669 | .009 | 0.288 | 2.453 | .016 |
| BDI | .196 | 1.650 | .103 | .165 | 1.359 | .178 | .066 | .521 | .603 | .160 | 1.253 | .214 | .270 | 2.277 | .025 |
| Full model | Adj R² |  |  | Adj R² |  |  | Adj R² |  |  | Adj R² |  |  | Adj R² |  |  |
|  | .239 |  |  | .208 |  |  | .144 |  |  | .127 |  |  | .246 |  |  |
| ª standardized coefficient  Results with p< 0.01 are considered significant (Bonferroni adjustment 0.05/5) | | | | | | | | | | | | | | | |
